# Understanding the Biology of Bone Sarcoma from Early Initiating Events through Late Events in Metastasis and Disease Progression

**DOI:** 10.3389/fonc.2013.00230

**Published:** 2013-09-17

**Authors:** Limin Zhu, Madonna M. McManus, Dennis P. M. Hughes

**Affiliations:** ^1^Department of Pediatrics – Research, UT MD Anderson Cancer Center, Houston, TX, USA

**Keywords:** osteosarcoma, Ewing sarcoma, metastasis, intravasation, neovascularization, tumor dormancy, anoikis resistance, cancer signaling

## Abstract

The two most common primary bone malignancies, osteosarcoma (OS), and Ewing sarcoma (ES), are both aggressive, highly metastatic cancers that most often strike teens, though both can be found in younger children and adults. Despite distinct origins and pathogenesis, both diseases share several mechanisms of progression and metastasis, including neovascularization, invasion, anoikis resistance, chemoresistance, and evasion of the immune response. Some of these processes are well-studies in more common carcinoma models, and the observation from adult diseases may be readily applied to pediatric bone sarcomas. Neovascularization, which includes angiogenesis and vasculogenesis, is a clear example of a process that is likely to be similar between carcinomas and sarcomas, since the responding cells are the same in each case. Chemoresistance mechanisms also may be similar between other cancers and the bone sarcomas. Since OS and ES are mesenchymal in origin, the process of epithelial-to-mesenchymal transition is largely absent in bone sarcomas, necessitating different approaches to study progression and metastasis in these diseases. One process that is less well-studied in bone sarcomas is dormancy, which allows micrometastatic disease to remain viable but not growing in distant sites – typically the lungs – for months or years before renewing growth to become overt metastatic disease. By understanding the basic biology of these processes, novel therapeutic strategies may be developed that could improve survival in children with OS or ES.

## Basics of Pediatric Bone Sarcomas

Osteosarcoma (OS) derives from primitive bone-forming mesenchymal cells and is the most common primary bone cancer (1). It occurs predominantly in growing adolescents and young adults, with a peak incidence at the age of 15–19 years. OS accounts for approximately 900 new diagnoses each year in the US, of which 15–20% patients present with overt lung metastases at initial diagnosis and about 40% patients develop metastases at a later stage (2, 3). Based upon the clinical outcomes of patients without overt metastasis at diagnosis during the pre-chemotherapy area, in which approximately 90% of patients developed lung metastasis 6–36 months later, it is presumed that the vast majority of apparently non-metastatic patients actually have micrometastatic disease at diagnosis. Current treatment including surgery, neoadjuvant, and adjuvant chemotherapy, has increased the overall survival rate for OS to around 70%. However, the clinical outcome for metastatic OS remains poor: fewer than 30% of patients presenting with metastases survive 5 years after initial diagnosis (4). More intensive chemotherapy protocols have not substantially increased long-term survival for patients with resistant or recurrent disease. Our knowledge of the mechanisms underlying OS metastasis is still limited. In order to improve the clinical outcomes for patients with poor prognosis, it is urgent to find new approaches to block metastasis in this disease.

Ewing’s sarcoma (ES) is the second most common bone malignancy in adolescents and young adults, after OS. This disease is most often characterized by a chromosomal translocation between chromosome 11 and 22, generating the EWS-FLI1 fusion gene (5). The protein encoded by this and other related EWS translocations acts as an aberrant transcription factor, driving the malignant behaviors of the transformed cell. ES has a peak incidence in the second decade of life, with a slightly higher rate in males (6, 7). Each year over 200 new cases are diagnosed in the United States. ES can develop at any site of the body, but it arises most frequently in bones and occasionally in soft tissues of the leg, pelvis, and chest wall (8). Similar to other pediatric sarcomas, nearly all mortality in patients with ES is caused by metastatic disease, not the primary tumor. Current therapy is directed toward treating both the primary tumor, since reducing tumor bulk prior to local control can result in better control, a less morbid procedure and better functional outcomes, and also at treating presumed microscopic metastasis. The intensive multimodal treatment with combination chemotherapy, surgery, and radiation has increased the 5-year event-free survival rate from less than 10% to over 70% (9–14).

The third most common type of primary bone malignancy is chondrosarcoma, which occurs mostly in adults and older teens. Chondrosarcoma is less well-studied than OS or ES, and there are few chondrosarcoma laboratory models in wide use. Since this condition is extremely rare in children, we will not address the biology of chondrosarcoma in this review.

## Bone Sarcoma Tumorigenesis

### Initiating events

Consistent with its high incidence in adolescents, OS preferentially develops in the growth plates of the most rapidly growing bones such as the distal femur and proximal tibia (15). A key driver of OS pathogenesis is thought to be over-activation of signal transduction pathways initiated by various growth factors such as insulin-like growth factor (IGF), transforming growth factor (TGF), and connective tissue growth factor (CTGF). Previous studies indicated that IGF-I, IGF-II, and their receptors are of vital importance to malignant growth of OS (16– 18). High expression levels of TGF-β1 in OS samples has been associated with a worse prognosis, and inhibition of TGF-β signaling severely hindered OS cell proliferation (19, 20). CTGF has been shown to exert its pro-tumorigenic effects on OS cells via integrin signaling (21).

A variety of genetic and chromosomal alterations also contribute to the generation of OS. Germline and somatic mutations of the retinoblastoma tumor suppressor gene (Rb) and p53 pathways are among the most common genetic changes found in OS (22, 23). Inactivation of the Rb pathway is observed in up to 70% of primary OS tumors (24). Retinoblastoma patients who have inherited Rb mutations are predisposed to OS (25). Experimental mice with both p53 and Rb inactivated in osteoblast progenitors or in limb-bud tissues develop OS (26, 27). While cyclin-dependent kinase inhibitor p16^INK4a^ acts upstream to promote Rb dephosphorylation and activation, deletion of this protein is detected in more than 10% of OS (28–31). The negative regulators of Rb, including cyclin-dependent kinase 4 (CDK4) and cyclin D1, have been found to be overexpressed in a small portion of high-grade OS (28, 30). The frequency of p53 mutations in OS is between 20 and 50% according to different reports and patients with Li–Fraumeni syndrome who carry inactivating mutations in p53 are at a much higher risk for developing OS than is the general population (32–34). MDM2, a major negative regulator of p53, is frequently amplified in OS (35–37). The p14^ARF^ protein which activates p53 function by inhibiting MDM2-mediated p53 degradation is absent in about 10% of OS cases (38). In addition to the alterations of major tumor suppressor pathways, oncogene activation is also centrally involved in OS tumorigenesis. Elevated expression of proto-oncogenes c-myc and c-fos could be detected in the majority of OS and is associated with poor prognosis (36, 39, 40). Forced overexpression of c-myc in bone marrow stromal cells derived from Ink4a/Arf null mice leads to genesis of OS (41). Coexpression of c-fos and c-jun enhanced OS formation in transgenic mice (42). The activator protein-1 (AP-1) is a heterodimeric transcription factor complex composed of c-jun and c-fos. Papachistou et al. displayed that AP-1 activity is associated with the pathogenesis and progression of OS (43). In addition, upregulation of Notch signaling components has been shown to contribute to the pathogenesis and invasiveness of OS (44, 45). Other molecular events that have been indicated in the tumor initiation of OS include the RECQ helicase pathways and the telomere maintenance mechanisms (46). These genetic alterations are summarized in Table 1.

**Table 1:** **Genetic alterations in osteosarcoma**.

Gene	Percent affected	Hereditary syndrome?	Murine model?	Reference
**TUMOR SUPPRESSORS**				
p53	20–50 (or more)	Li–Fraumeni	Yes	McIntyre et al. (32), Lonardo et al. (37), Gokgoz et al. (33), Hauben et al. (34)
Rb	Up to 70	Retinoblastoma	Yes	Eng et al. (25)
p16^INK4A^/p14^ARF^	∼10%	Dysplastic nevus syndrome	Only with AP-1 or c-myc overexpression	Lopez-Guerrero et al. (38), Shimizu et al. (41)
**ONCOGENES**				
MDM2	6–14	SNP309 of MDM2 have accelerated tumor formation	Not for OS; roles for other malignancies	Bond et al. (227)
AP-1 (c-jun/c-fos)	40–60 for both c-fos and c-jun	None known	Yes	David et al. (228)
Notch	Unknown	No	Upregulated in p53/Rb models of OS	Engin et al. (44)

The vast majority of ES is characterized by the specific chromosomal translocation which fuses the EWSR1 gene on chromosome 22 and the FLI1 gene on chromosome 11 to form the chimeric EWS-FLI1 oncogene. It is believed that the EWS-FLI1 fusion protein is the master regulator of tumorigenesis in ES. It functions as a transcription factor and modulates multiple signaling pathways including Hedgehog/GLI, Wnt/β-catenin, IGF1/IGF1R and TGF-β, and Notch/p53 (47–52). According to previous studies, c-Myc, GLI1, cyclin D1, Cav-1, VEGFA, IGF1, NKX2-2, AURKA, EZH2, and NR0B1 are among the downstream targets of EWS-FLI1 that are upregulated in ES to promote cell survival and proliferation (53–56). Tumor suppressor genes such as NOTCH, p53, p21^WAF/Cip1^, p27^Kip1^, p57z, TGFBR2, IGFBP3 are downregulated by EWS-FLI1 to protect cells from growth arrest, senescence, and apoptosis (55). In addition to the formation of EWS-FLI1, other genetic alterations also contribute to pathogenesis of ES. Like OS and many other human cancers, p53 mutations have been detected in a small fraction of ES cases (57, 58). Amplification of MDM2 and Ras are also commonly found in ES tumor samples (59, 60). The CDKN2A locus where p14^ARF^ and p16^INK4a^ are located is lost in 15–30% of ES and is associated with poor clinical outcomes (60, 61).

### Neovascularization

The process of blood vessel formation, or neovascularization, is comprised of angiogenesis and vasculogenesis. Tumor angiogenesis is the extension of blood vessels from preexisting vascular structures, while vasculogenesis is the *de novo* formation of vessel networks through the recruitment of bone marrow-derived precursor cells. Neovascularization is essential for sustained tumor growth and provides the systemic network that stimulates metastasis. Without the formation of supporting vasculature, tumor cells would be unable to obtain the nutrients and oxygen necessary for proliferation and would not be able to mediate metastatic spread. A delicately controlled balance between pro- and anti-angiogenic factors typically regulates angiogenesis; environmental stressors or genetic changes like hypoxia, acidosis, oncogene activation, and loss of tumor suppressor genes lead to dysfunction of this balance and result in angiogenesis.

Hypoxia-inducible factor-1 (HIF-1) is a key transcription factor that regulates the expression of genes responsible for the survival and adaptation of cells as they move from normoxia (∼21% O_2_) to hypoxia (∼1% O_2_). HIF-1 is made up of an oxygen related α subunit (HIF-1α) and a constitutive β subunit (HIF-1β) (62). The stability of HIF-1α is regulated by prolyl hydroxylase domain proteins (PHDs), while its transcription is regulated by factor inhibiting HIF (FIH). In normoxic and mildly hypoxic conditions, PHDs hydroxylate HIF-1α, resulting its association with von Hipper–Lindau (pVHL) ubiquitin E3 ligase complex allowing for rapid proteasomal degradation of HIF-1α (63–65). Under the extreme hypoxic conditions within a tumor, HIF-1α is stabilized and binds to the promoter region of VEGF where it mediates its upregulation (66). This signaling cascade can take place in both tumor cells and the non-malignant cells – tumor associated endothelial cells, etc. – that are found in the hypoxic center of tumors (67).

VEGF has been shown to be upregulated by a number of other factors that are released in response to the rapid proliferation of tumor cells; these include transforming growth factor α (TGF-α), fibroblast growth factor 2 (FGF-2), and hepatocyte growth factor (HGF) (68). Upregulation of VEGF also can be mediated by the transcription factor Wilms tumor protein 1 (WT1) (69). As the activation of growth factor receptors like EGFR and Integrin lead to Src activation, Ras/MAPK signaling and activation of the transcription factor STAT3 are initiated, allowing for cell cycle progression and proliferation (70, 71). STAT3 signaling is required for VEGF production and its activation results in a positive feedback loop that further increases the production of FGF and VEGF, leading to the increased induction of vascular permeability and neovascularization (72). Thus signaling by EGFR or other ERBB family kinases is upstream of VEGF release in most cases.

**Figure 1:**
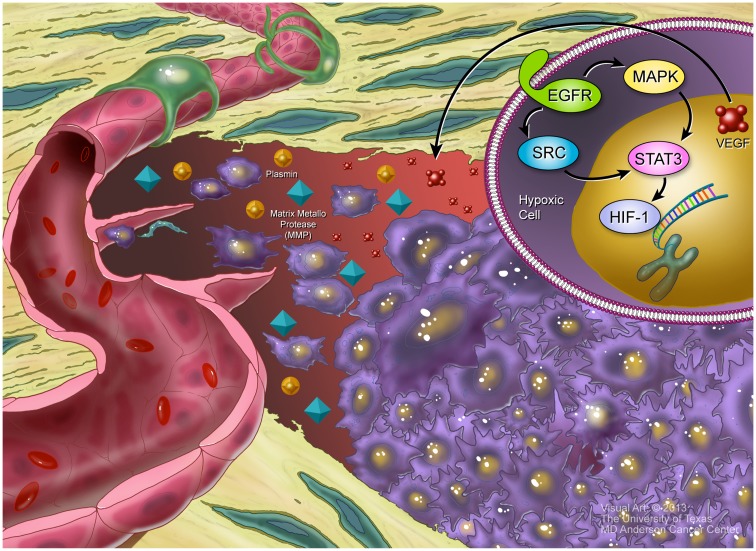
**Hypoxia and angiogenesis**. Inset, upper right: HIF1a has been stabilized in the hypoxic center of a tumor, allowing it to bind to the VEGF promotor. Additionally, EGFR signaling, acting through SRC and the MAPK cascade, induce STAT3, which helps promote VEGF transcription. Larger picture: the hypoxic tumor, in purple, releases VEGF that establishes a gradient from the tumor to the nearby blood vessel. VEGF stimulates release of MMPs and plasmin, which help mediate digestion of extracellular matrix proteins, facilitating migration of both endothelial cells and tumor cells. In response to the VEGF gradient, some endothelial cells acquire a “tip cell” phenotype, pushing long extensions toward the tumor. These tip cells will eventually form the new blood vessels that provide a blood supply to the growing tumor.

VEGF is the best characterized pro-angiogenic factor and is considered the most important factor involved in the development of the vasculature. There are a number of different VEGF molecules (VEGFA through VEGFE) that bind to VEGF receptors (VEGFR1-3). VEGFA binds to VEGFR2 and initiates a number of divergent signaling pathways (73). Among the proteins that are upregulated upon VEGF activation are the matrix metalloproteinase (MMP) and plasmin proteases (74), which act on the vascular network by breaking down the extracellular matrix (ECM) and allow for tumor cell invasion, as well as the migration of the precursor cells that give rise to vascular structures: pericytes and endothelial cells. These events are depicted in Figure 1. Additionally, VEGF signaling also induces the expression of the anti-apoptotic factors Bcl-2 and survivin (75), as well as the ERK/NF-kB and PI3K pathways (76). These effectors promote tumor cell proliferation and survival.

VEGF has been shown to be amplified in human OS (77). Elevated VEGF expression in OS has been associated with the development of lung metastases, compared to patients with VEGF-negative tumors (82 vs. 10% respectively), and VEGF-positive OS tumors have been shown to have significantly worse overall and disease-free survival rates (78–80). Patients with ES have increased circulating VEGF levels compared to controls (79, 81, 82). Interestingly, EWS-ETS fusion oncoproteins drive the expression of VEGF in an SP1 dependent manner (83) and may contribute to the increased VEGF levels observed in patients. Bone marrow driven vasculogenesis is essential for tumor growth in ES (84). The upregulation of pro-angiogenic factors like VEGF, FGF, TGF-α, HGF, platelet-derived growth factor (PDGF), angiopoietin 1 (Ang1), and ephrin-B2 combined with the down-regulation of anti-angiogenic proteins like thrombospondin-1, TGF-β, troponin I, pigment epithelial-derived factor (PEDF), and reversion-inducing cysteine-rich protein with Kazal motifs (RECK) allows for rapid neovascularization (68, 85–88).

### Intravasation

**Figure 2:**
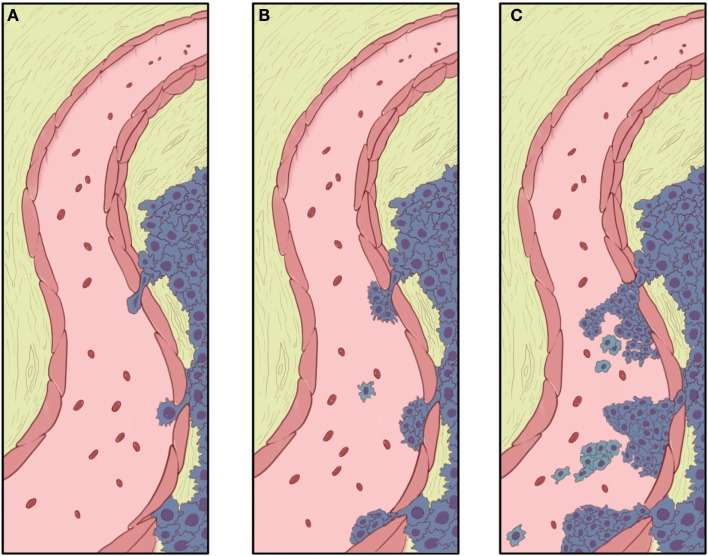
**Invasion and intravasation**. **(A)** The growing tumor advances to the basement membrane of a nearby blood vessel, with the proteolytic functions of MMPs and plasmins clearing a pathway and allowing the leading cells to approach (bottom) and eventually push between (middle and top) the endothelial cells. With mass migration, other tumor cells may follow behind the leading cells. These trailing cells do not necessarily have as much invasive capacity as the lead cell, but may have their invasion and intravasation facilitated by the more invasive lead cells. **(B)** Migrating tumor cells effectively form a small “beachhead” in the spaces where lead cells have pushed between and through gaps in the endothelial cells. **(C)** With further mass migration, tumor cells begin obstructing the flow of blood through the vessel, and encounter red cells, platelets, and other blood components. A tumor thrombus may occlude blood flow and, is the vessel is large enough, cause symptoms of deep venous thrombosis. Clumps of tumor and individual tumor cells detach from the main mass of tumors and are carried away in the blood stream. Individual cells would be subject to anoikis when loose in the blood stream. Some cells in tumor clumps may be protected from anoikis by their attachment to other cells in the clump.

The first step in metastasis is migration from the primary tumor site and invasion through the basement membrane, allowing metastatic cells to enter circulation and disseminate distantly. These events are depicted in Figure 2. Since pulmonary metastasis is the major cause of death in both OS and ES, elucidating the mechanisms controlling metastasis is vital for improving patient outcomes. By identifying the molecular alterations associated with cell migration and invasion, it is hoped that novel therapies could be developed to prevent metastases.

Degradation of the ECM, facilitated by the action of MMPs, is a prerequisite of tumor invasion and metastasis in a variety of human cancers including OS and ES. MMP-2 and MMP-9 in particular have been repetitively implicated in OS cell invasion (89–91). In addition, m-calpain, an intracellular protease that modulates cell adhesion and motility, plays a pivotal role in promoting cell invasion in OS. Fan et al. demonstrated that the inhibition of m-calpain and the concomitant blockade of MMP-2 secretion led to a strong decrease in OS cell adhesive and invasive ability (92). The urokinase plasminogen activator (uPA) and its receptor uPAR are important mediators of OS cell invasion through activation of plasminogen and pro-MMPs (93). Down-regulation of uPAR prohibited cell adhesion, migration, and invasion without affecting cell proliferation *in vitro*, resulting in a marked reduction of pulmonary metastases *in vivo*.

The Wnt/β-catenin pathway and its antagonists are involved in multiple facets of tumor progression including invasion and metastasis. High levels of β-catenin and aberrant activation of Wnt signaling are frequently observed in OS samples and such molecular alterations are correlated with the incidence of metastasis (94, 95). Evidence from multiple sources suggests that the Wnt/β-catenin pathway promotes metastasis in OS through modulation of Wnt target genes including MMP-9, cyclin D, c-myc, and survivin (96). Various approaches to disrupt Wnt signaling have been proven effective in inhibiting cell migration and invasion in laboratory models (96, 97). Wnt signaling also may mediate activation of Notch pathway signaling, which also has been implicated in OS pathogenesis and metastasis. The Notch receptor 1, 2, and the downstream target gene Hes1 is upregulated in highly metastatic OS cells (44, 98), and inhibition of Notch signaling by either molecular or pharmacologic means reduced both OS proliferation and metastasis (44).

Src signaling is another pivotal pathway that contributes to the aggressive phenotype of OS cells. Pharmacologic inhibition of the c-Src-mediated signaling pathway suppressed phosphorylation of focal adhesion kinase (FAK) and activation of other downstream proteins, resulting in significantly decreased cell migration and invasion in a panel of OS cells (70, 99). However, the therapeutic effect of Src inhibition is very limited in animal models (70), suggesting that Src is not an essential mediator of metastasis in OS, or that “rescue pathways” exist to activate FAK and other Src targets.

Recent studies in OS have uncovered some novel targets for anti-metastatic strategies. Parathyroid hormone (PTH), PTH peptides, as well as the PTH receptor (PTHR) have been shown by multiple reports to enhance OS cell migration and invasion (100, 101). Autocrine motility factor (AMF), a major cell motility-stimulating factor secreted by tumor cells, is associated with tumor metastasis in various human cancers including OS (102). Suppression of AMF expression induced mesenchymal-to-epithelial transition (MET) in OS and reduced cell motility and invasiveness (103). Tissue microarray analysis revealed that cysteine-rich protein 61 (CCN1/Cyr61) is overexpressed in most cases of OS when compared to normal bone tissue (104). The same study showed that genetic manipulation of Cyr61 expression levels in OS cells concomitantly influenced cell invasion and migration *in vitro* and metastatic potential *in vivo*. Interleukin-6 (IL-6) is a multipotent cytokine which has been implicated in the progression of multiple human malignancies including OS. Lin et al. demonstrated through a series of IL-6 knockdown experiments that interaction between IL-6 and its receptor IL-6R is required for OS cell migration via the activation of intercellular adhesion molecule-1 (ICAM-1) expression (105).

Similar to their role in OS, MMPs are essential mediators of ES cell migration and invasion as they are responsible for the localized degradation of ECM components, which serve to clear a pathway for the invading ES cells. Caveolin-1 (CAV-1) controls ES cell migration, invasion, and lung colonization by upregulation of secreted protein acidic and rich in cysteine (SPARC), which in turn activates MMP-2 and MMP-9 (106). Studies on ES patient samples have established a link between the expression of CCN-3 which is a secreted, ECM-associated signaling protein, and a higher risk to develop lung metastases (107, 108). Ectopic expression of CCN-3 in ES cells reduced cell proliferation rate and anchorage-independent growth while promoting cell migratory and invasive capabilities, which could be due to the decreased expression of α2β4 integrin receptor and increased cell surface localization of MMP-9 (109). Hauer et al. identified DKK-2 as a pro-metastatic gene highly overexpressed in a panel of ES cell lines (110). By regulating the activity of MMP-1, DKK-2 increased ES cell invasiveness and metastatic potential. The same study also demonstrated that DKK-2 overexpression enhanced bone invasiveness and osteolysis, both of which facilitate the metastatic spread of ES. A DNA microarray analysis of 11 ES samples and 133 normal tissues revealed that the six-transmembrane epithelial antigen of the prostate 1 (STEAP1), a membrane-bound mesenchymal stem cell marker, is a signature gene highly expressed in ES (111). Further study on this gene demonstrated that STEAP1 promotes proliferation, anchorage-independent growth, tumorigenicity, and metastasis in ES cells (112). In addition, STEAP1 overexpression is associated with elevated reactive oxygen species (ROS) level in ES, which could also promote tumor aggressiveness. The histone methyltransferase enhancer of Zeste, Drosophila, Homolog 2 (EZH2) is another important mediator of ES tumor growth and metastasis, driven by EWS/FLI1 (54). Down-regulation of EZH2 significantly impeded tumor growth and inhibited lung and liver metastasis *in vivo*.

Other signaling pathways, such as Src and Wnt signaling, are also of great importance in ES cell migration and intravasation. Constitutive activation of Src kinase has been observed in ES metastasis (99, 113). Shor et al. demonstrated that inhibition of Src phosphorylation and its downstream targets, including FAK and Crk-associated substrate (CAS), by dasatinib blocked *in vitro* cell migration and invasion (99). Overexpression of C-X-C chemokine receptor type 4 (CXCR4) correlates with ES metastases (114, 115). Upregulation of CXCR4 by the non-canonical Wnt family member Wnt5a has been shown to promote ES cell migration in absence of Wnt antagonist SFRP5 (115). Landuzzi et al. reported a positive role of stem cell factor (SCF) and its receptor c-kit in promoting ES metastasis (116). They have demonstrated in multiple ES cell lines that SCF, a growth factor abundantly expressed in ES and the tumor microenvironment, induced a strong increase in cell motility via activation of c-kit. A recent publication also indicated that overexpression of ERBB-4, which is observed in a panel of ES cell lines and metastatic tumor samples, led to increased invasion and migration *in vitro*, as well as enhanced metastatic capacity *in vivo* through activation of PI3K-Akt, FAK, and the Rac1 GTPase (117).

Specific regulation of microRNA expression also may play a role in regulating intravasation and metastasis. MicroRNAs (miRNAs) are a class of endogenous non-coding small RNAs that contains about 18–25 nucleotides. miRNAs regulate translational suppression or cleavage of their target mRNAs through perfect or imperfect binding to the 3′-untranslated region (3′-UTR) of their target mRNA. Deregulation of miRNAs has been observed in many human cancers, and recent reports indicate the involvement of miRNAs in development and metastasis of bone sarcomas. Ziyan et al. identified a pro-metastatic function of miR-21 by comparing tumor samples and matched normal bone tissues (118). miR-21 promotes the activity of matrix MMPs by negatively regulating the tumor suppressor gene reversion-inducing cysteine-RECK, and knockdown of miR-21 decreased cell migration and invasion. miR-27a is another pro-metastatic miRNA validated by Jones and colleagues after they surveyed a panel of OS cell lines together with normal osteoblast cells (119). On the contrary, miR-143 stood out as an anti-metastatic miRNA when the miRNA expression profiles were compared between HOS cells and its metastatic subclone 143B cells (120). Reintroduction of miR-143 into the highly metastatic cells suppressed invasiveness and impeded pulmonary metastasis through inhibition of MMP-13. Expression of miR-183 also correlates with OS pulmonary metastasis (121). Suppression of miR-183 led to elevated ezrin level and activation of p-p44/42, resulting in enhanced metastatic ability of OS cells. Mao et al. found that miR-195 plays a similar role in OS metastasis (122). With the fatty acid synthase (FASN) as a direct target, miR-195 significantly inhibited OS invasion and migration in USOS cells.

### Anoikis resistance

After tumor cells detach from their primary site and enter into the bloodstream, the absence of cell–cell adhesion, and cell-ECM interaction can trigger specific cellular apoptosis, a process termed “anoikis.” Therefore, acquisition of resistance to anoikis is a critical step in survival and expansion of metastatic cells. Multiple molecular pathways contribute to anoikis evasion in OS and ES, including integrin signaling, PI3K/Akt, Src, Wnt/β-catenin, and the BcL family (123). Since anoikis resistance is an essential step in tumor progression, elucidation of the molecular mechanisms underlying this process may provide additional therapeutic strategies for targeting metastasis.

Integrins, a family of cell adhesion receptors that can activate multiple signal transduction pathways, play a key role in the survival of tumor cells in anchorage-independent environments. Several studies have indicated a correlation between integrin expression and OS metastasis (124, 125). Wan et al. demonstrated that β4 integrin is highly expressed in OS cell lines and tumor samples (125). Knockdown of β4 integrin suppressed cell proliferation in anchorage-independent conditions without affecting cell growth in adherent cultures *in vitro* and effectively inhibited pulmonary metastasis *in vivo*. In addition, β4 integrin has been found to interact with ezrin as a way to maintain its expression level in OS cells. Marco et al. reported that α4 integrin expression also confers resistance to anoikis in OS cells (124). They demonstrated that α4 integrin is abundantly expressed in metastatic OS lesions. Blocking α4 integrin with a monoclonal antibody increased cell death in suspended cells but not in adherent cells.

Another way for tumor cell to avoid anoikis is to alter the expression pattern of integrin subunits, which leads to elevated PI3K/Akt signaling and decreased cell apoptosis (126). One study revealed that Src-dependent activation of the PI3K/Akt pathway, which is independent of FAK phosphorylation, is essential for OS cells to gain anoikis resistance (127). Pharmacological inhibition of Src or PI3K activity restored sensitivity to anoikis in OS cells. Cantiani et al. also confirmed the role of Src in mediating anoikis resistance by showing that Cav-1, a tumor suppressor gene significantly downregulated in OS cell lines and tumor samples, inhibited migration, invasion, and anchorage-independent growth of OS cells by blocking Src family kinase activity and Met signaling (128). Beristain et al. indicated that the receptor activator of NF-κB (RANK) signaling pathway also protects OS cells from anoikis (129). Activation of RANK enhanced cell survival in anchorage-independent environments while knockdown of endogenous RANK inhibited the tumorigenic ability of several mouse OS cell lines. Other survival mechanisms adopted by solitary OS cells to evade anoikis include activation of the Wnt/β-catenin pathway and overexpression of anti-apoptotic genes such as Bcl-2. Lin et al. demonstrated that upregulation of Bcl-2 protected detached OS cells from anoikis by inhibiting activation of caspase-8, which can induce apoptosis when cell adherence is disrupted (130). High levels of Wnt family proteins have been reported in OS patient samples, and constitutive Wnt activation is associated with high metastatic potential (94). Rubin et al. showed that overexpression of the Wnt inhibitory factor-1 (WIF-1) significantly decreased anchorage-independent growth and cell motility in 143B cells and inhibited *in vivo* tumorigenesis and metastasis (97).

**Figure 3:**
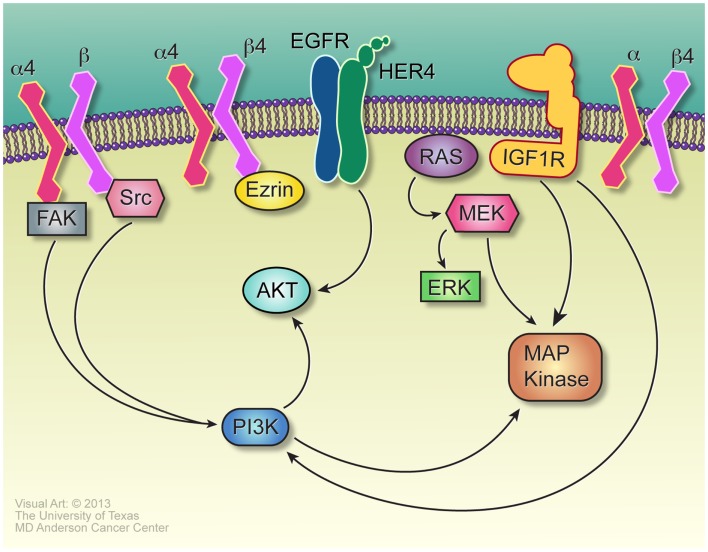
**Signaling in anoikis resistance**. The α4 and β4 integrins, paired either with each other or with other integrins, initiate signals vital to anoikis resistance, through interaction with Ezrin in the case of β4, and Src for α4. Src-mediated activation of PI3K may be independent of its well-described role in activating FAK. Her-4, induced by E-cadherin, signals strongly through AKT and mediates anoikis resistance, certainly for Ewing sarcoma and probably in osteosarcoma as well. While many receptor tyrosine kinases may mediate Ras activation and Ras-dependent and – independent activation of the MAP kinase cascade, IGF1R is an especially important source of these signals, especially for anoikis resistance in Ewing sarcoma.

Some of the molecular pathways mediating anoikis resistance in ES are similar to that of OS. Sustained activation of PI3K/Akt and Ras/ERK pathways in ES cells has been indicated as an essential process for anchorage-independent cell survival (131). PI3K inhibition markedly reduced anchorage-independent growth of TC32 cells through regulation of the cyclin D1 level. Aberrant activation of the MEK/MAPK pathway contributes to the survival of detached ES cells (132). Blockade of the MEK/MAPK pathway by specific inhibitors impaired cell motility and colony formation ability *in vitro*. The insulin-like growth factor receptor (IGF-1R), a receptor tyrosine kinase upstream of PI3K/Akt and MAPK pathways, is another important signaling pathway involved in anoikis resistance in ES (133). IGF-1R is highly expressed in both ES cell lines and patient tumor samples and inhibition of the IGF-1R signaling resulted in profoundly reduced cell migratory ability and increased anoikis-induced apoptosis in several ES cell lines. Additional pathways also may mediate anoikis resistance in ES. Douglas et al. demonstrated that the polycomb gene BMI-1 is highly expressed in ES cells and it promotes anchorage-independent growth and tumorigenesis by activating downstream targets which modulate cell adhesion pathways (134). Tirado et al. showed that, unlike its tumor suppressive role in OS, Cav-1 is highly expressed as an oncogene in ES cell lines and tumor samples (135). As the direct transcriptional target of EWS-FLI1, Cav-1 promotes the malignant phenotype of ES cells. Knockdown of Cav-1 impeded anchorage-independent growth *in vitro* and tumorigenesis *in vivo*, which is associated with suppression of E-cadherin and upregulation of Snail. E-cadherin belongs to a class of transmembrane proteins that regulate cell–cell adhesion. As ES cells readily form multicellular spheroids under detachment conditions, a concomitant upregulation of E-cadherin has been observed. Kang et al. reported that E-cadherin protects ES cells from anoikis-induced apoptosis through downstream activation of Akt in an ERBB-4 dependent manner (136). Several pathways relevant to anoikis resistance are depicted in Figure 3.

### Extravasation and attachment

To form metastases, disseminated tumor cells (DTCs) that survive circulation must extravagate into foreign tissues by attaching and adapting to the metastatic microenvironment. DTCs are significantly larger than normal blood cells; this causes the formation of microembolisms that can get trapped in capillary beds throughout the body (137). Interestingly, these microembolism do not spread indiscriminately. Rather, they tend to appear in a small number of highly specific target organs, which can vary depending on the histology of the original tumor. Over 80% of all metastases in OS occur in the lungs (138). Most ES metastases also occur in the lungs, but ES also spreads frequently to the bone and bone marrow (139). This suggests that there are specific environmental cues that allow for the survival and growth of DTCs. These environmental factors that make specific organ sites favorable to DTC metastases rely on the interactions between specific molecules expressed on both the DTCs and the endothelium of the target organ (76).

Chemokines and proteinases mediate distant colonization (76, 140). Chemokines and proteinases are responsible for the organ-specific localization of DTCs and the extravasation of DTCs into foreign tissues, respectively (140–145). In sarcomas, chemokines bind to G-protein coupled receptors on the plasma membrane of tumor cells in the lungs (115, 141, 142, 144, 146, 147). CXCR4 is a highly expressed chemokine in OS and is implicated as the most important contributor in the development of site-specific metastases. Binding of CXCR4 to C-X-C motif chemokine ligand 12 (CXCL12), which is abundantly expressed in the lungs, allows for the adhesion and extravasation of OS cells into the lung (140–144, 146, 148, 149). High CXCR4 expression in OS patient samples is inversely correlated with event-free, overall, and metastasis-free survival (142). Although inhibition of CXCR4 binding to CXCL12 did not consistently inhibit the development of tumor metastases (148), targeting CXCR3 *in vivo* to inhibit its binding to ligands CXCL9, 10, and 11 did inhibit OS lung metastases (149). In ES CXCR4 expression correlated with Wnt5a expression and was significantly higher in patients that presented with metastasis at diagnosis, compared with patients without metastases, and also correlated with poor survival (115). Selectins and integrins also facilitate the adhesion of malignant sarcoma cells to the endothelial lining of the lungs (150).

Ezrin is a membrane cytoskeleton linker protein that mediates membrane organization and cell microenvironment interactions (151) and is also believed to facilitate OS-DTC anchorage to the lung tissue. High Ezrin expression is correlated with: metastasis in animal models of OS (152–154), higher risk of metastatic relapse, and poor survival in pediatric OS patients (153, 154). Ezrin is thought to promote the formation of metastases through β4 Integrin mediated activation of the PI3K, Akt, and MAPK pathways and thus Ezrin stimulates survival and proliferation of the DTC in the lung (76, 125, 146, 153, 154). Inhibition of Ezrin-activated MAPK/Akt signaling with Sorafenib suppresses the development of lung metastases in laboratory models (155).

The nuclear factor-kappa B (NF-κB) is known to be involved in the development of lung metastases (156–159). The linear ubiquitin chain assembly complex (LUBAC) activates both NF-κB and intercellular adhesion molecule-1 (ICAM-1), and is necessary for the extravasation and retention of DTCs in the lungs (160). Therefore, the knockdown of LUBAC is able to decrease both the number and the size of the metastatic nodules within the lungs of mice injected with OS cells (160).

### Dormancy

For patients who present without radiographic evidence of metastasis, pulmonary metastases often become evident only 6 months to 3 years after diagnosis, long after the resection of the primary site. This suggests that as cells disseminate, extravagate, and attach to the lung environment, they undergo growth arrest and become dormant. Despite the clinical significance of this dormancy, the mechanisms underlying it and the outgrowth of macrometastatic lesions from these dormant micro-metastases remain poorly understood. Some evidence suggests that a subpopulation of cancer cells exhibit stem-like properties which are capable of disseminating to distant organs (161–165). These cancer stem-like cells (CSCs) are purported to have the ability to self-renew and populate a growing tumor, an increased capacity for DNA repair, and higher expression levels of anti-apoptotic proteins than differentiated cells (166–170). These properties provide CSCs with the ability to survive for long periods of time under metabolic and environmental stresses (e.g., hypoxia) while they arm tumor cells with ATP-binding cassette (ABC) transporters that actively efflux chemotherapeutics from target cells, thus providing tumor cells with increased drug resistance (171–176).

Two additional explanations for tumor dormancy focus on the senescence of either multiple cells (*tumor mass dormancy*) or individual cells (*cellular dormancy*). *Tumor mass dormancy* is the process by which the cells that make up micro-metastases are balanced between proliferation and apoptosis, and accordingly the mass does not grow (177, 178). This process purportedly relates to the lack of nutrients and oxygen from the vasculature (179–183). *Cellular dormancy* cites the process by which individual tumor cells enter a quiescent state and do not divide anymore (177, 184, 185). Such cells are typically more resistant to conventional drugs because current treatments tend to target dividing cells. The two processes, of course, are not mutually exclusive, as small foci of tumors may contain individual cells that are growth arrested but not terminally differentiated.

In order to elucidate the cellular mechanisms that define metastatic dormancy, Almog et al. designed an OS *in vivo* model for dormancy and performed gene expression analysis of cells in the dormant state versus cells in the proliferative state (186). Since tumor growth is dependent on vascularization, metastatic dormancy is generally associated with the upregulation of anti-angiogenic proteins such as angiomotin, which suppresses tumor growth while maintaining the dormant state of DTCs (179). Almog et al. observed a marked increase in the upregulation of anti-angiogenic proteins in dormant cells (186). Administration of recombinant angiogenic factors was shown to allow the transition from anti-angiogenic dormancy, to pro-angiogenic tumor growth (187). Additionally, an upregulation of endocan in rapidly proliferating cells was observed, suggesting that poor connection to the ECM of the pulmonary environment may also be associated with dormant micro-metastases (186) while proper anchorage to the ECM would stimulate cells to proliferate via β1-integrin signaling (183). The anti-apoptotic protein Bcl-xL, α5β1-integrin mediated activation of NF-κB, and the ratio between ERK and p38-MAPK proteins all appear to be involved in the regulation of tumor dormancy (76, 146, 182, 183). The role of anti-angiogenic signals in maintaining dormancy and pro-angiogenic signals in promoting renewed growth of pulmonary micro-metastases may explain the clinical observation, often repeated by patients and families, that pulmonary metastases are often identified within a few months after a major operation. Patients often tell us that “operations spread the sarcoma.” The more probable truth is that major operations, and the cytokines released as a result of them, awaken dormant micrometastatic lesions.

As of now, we are unaware of publications that describe dormancy in ES. Given that ES patients do experience recurrence after long periods of disease-free survival, it seems probable that systems of dormancy do occur for ES. Whether these systems are mechanistically similar to dormancy in other sarcomas remains to be shown.

## Chemoresistance

Tumor resistance to chemotherapy has always been a major obstacle for treating patients with bone sarcomas. Intrinsic resistance to chemotherapy has been detected in a small portion of patients. More commonly, however, patients present with disease that initially is responsive to cytotoxic therapy, but acquires resistance that is evident when tumors recur. Thus, a better understanding of the molecular mechanisms underlying the development of chemoresistance will facilitate the discoveries of novel treatment strategies to overcome this resistance and improve patient outcomes.

Most studies of chemoresistance in bone sarcomas thus far have focused on molecules and pathways already known to effect sensitivity to chemotherapy in more common tumors. Increased levels of P-glycoprotein, a transmembrane ATP-dependent efflux pump which plays a key role in multidrug resistance, is strongly associated with poor response to chemotherapy and reduced survival in both OS and ES patients (188–191). As an important predictive factor for chemoresponse in many other cancers, Her-2/ERBB2 has been extensively studied in bone tumors, yet the role of Her-2 overexpression in the development of chemoresistance in OS and ES remains controversial and ambiguous. Some studies suggested that Her-2 expression is significantly correlated with histologic responses to preoperative chemotherapy while other studies indicated that Her-2 expression in OS and ES has no prognostic significance (191–195). Our group has shown that expression of Her-4/ERBB-4 confers a worse prognosis for neuroblastoma by mediating reduced cell proliferation and anoikis resistance, as well as conferring direct resistance to cisplatin, doxorubicin, and ifosfamide (196). Similar studies to evaluate the role of ERBB-4 in OS are ongoing in our lab, and a role for ERBB-4 in ES has been established (136). The Bcl-2 family proteins, which mediate various steps of apoptosis, are important regulators of chemotherapy-induced cell death. The anti-apoptotic proteins Bcl-2 and Bcl-xL are frequently overexpressed in OS and ES and are associated with development of resistance to a variety of chemotherapeutic agents or radiation (197–201). Up- and down-regulation of these proteins can significantly reduce or enhance the chemosensitivity of OS and ES cells. Overexpression of EWS/FLI-1 direct target genes, CAV-1 and glutathione *S*-transferase M4 (GSTM4), also help modulate resistance to chemotherapy in ES cells (202, 203). In addition, miRNA has been found to play a vital role in conferring chemoresistance in OS and ES cells. Song et al. indicated that miR-140 and miR-215 are both involved in OS chemoresistance by inducing a decreased cell proliferation through cell cycle arrest at G1 and G2 phase respectively (204, 205). The miRNAs miR-34a, miR-708, and miR-125b are essential miRNAs that regulate cell survival and chemosensitivity of ES cells (206–208). Analysis of 34 ES tumor samples revealed that reduced or absent expression of miR-34a, which is associated with p53 inactivation, predicted a worse clinical outcome and restoration of miR-34 activity *in vitro* markedly increased ES cell sensitivity to doxorubicin and vincristine (206). Similarly, repression of miR-708 in ES tumor samples is associated with up-regulation of the DNA repair protein and transcriptional cofactor EYA3, which directly led to enhanced cell survival and resistance to DNA-damaging chemotherapeutics (207). On the contrary, miR-125b has been shown to promote multidrug resistance in ES cells by suppressing the expression of pro-apoptotic proteins, p53 and Bak. Knocking down miR-125b in the chemo-resistant ES cells increased their sensitivity to doxorubicin, etoposide, and vincristine (208).

## Evasion of Immune Surveillance

The survival of DTCs and, to a lesser extent primary tumors, depends on the evasion of the host immune system. Faulty regulation of a number of key genes that control the immune system allow DTCs to achieve a survival advantage. The down-regulation of HLA class 1, a cell surface receptor that impairs the recognition of tumor cells by the host cytotoxic T-lymphocytes, is one such mechanism (209, 210), and expression of HLA class I has prognostic significance for OS (209). DTCs can also induce the production of IL-10 expression in OS, resulting in immunosuppression (146, 211). Additionally, corruption of the Fas pathway or low expression of Fas on the cell surface of OS and ES cells inhibits the ability of cytotoxic natural killer cells to detect and clear DTCs, thus yielding an increase in metastatic potential. OS metastatic tissue samples are Fas negative (212, 213), and low Fas expression correlates with disease progression and poor survival (212, 214–217). Despite these observations, adoptively transferred T cells did show some clinical benefit in one pilot study (218). The use of interferon in OS patients has been studied, though the results of the Euramos study – the largest clinical evaluation of interferon for OS to date, have still not been reported as of the time of this review.

The innate immune system may offer greater promise for treating OS and ES. The macrophage-activating agent L-MTP-PE, first evaluated by Kleinerman and colleagues, reduced metastatic tumor burden in experimental laboratory models (219), and had proven benefit in dogs with spontaneously occurring OS (220). After a series of smaller trials, a cooperative group study of L-MTP-PE demonstrated an 8% improvement in overall survival in patients treated with L-MTP-PE (221), though the initial studies were difficult to interpret due to an interaction with ifosfamide that has not been fully characterized (222). The benefit was greatest for patients who presented with metastatic disease that were able to achieve a complete remission through surgical resection (26). This drug, now with the generic name mifamurtide, has been given regulatory approval by the EMA, Mexico, Japan, Israel, and several other countries, but has yet to be accepted by the FDA. A similar agent, Imm-Ther, was studied in ES, but the data from that small study have not been reported.

**Figure 4:**
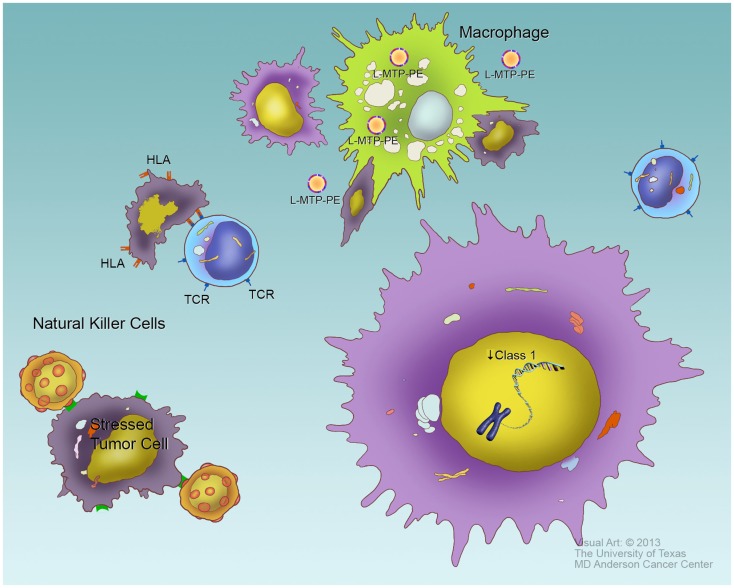
**Evasion of immune response**. To survive, tumor cells must evade several components of cellular immunity. Cytotoxic T cells, through their T cell receptors (TCR), recognize foreign antigens, and kill cells bearing these antigens presented by class I HLA. Tumor-specific antigens can potentially be treated as foreign by the immune system. Tumor cells, such as the one in the lower right, typically downregulate HLA expression as a means of evading T cell response. Natural killer (NK) cells (left side of figure) recognize specific surface molecules expressed on “stressed” cells, especially tumor cells. One requirement for full NK response is the absence of self-HLA, so the down-regulation of HLA that allows tumors to evade cytotoxic T cells may facilitate their recognition by NK cells. Macrophages may ingest and destroy tumor cells in an antigen non-specific manner, though this process may require macrophage activation such as typically occurs in the sites of infection. The macrophage-activating drug L-MTP-PE has been shown to induce macrophage activation (top of figure) and improves survival in high-risk osteosarcoma patients when given in first complete remission or at a time of minimal residual disease, presumably by increasing phagocytosis on tumor cells.

Natural killer cells (NK cells) may be another option for harnessing immune responses against OS and ES. Multiple laboratory studies demonstrated the ability of NK cells to recognize and kill OS cells (223–226). The regulation of NK cell cytolytic activity is complex, and includes both activating and inhibitory receptors, as well as induced tolerance to specific self-receptors. These mechanisms have been reviewed recently (227). One aspect on NK recognition, however, can be described relatively simply: the presence of “self-HLA Class I” has an inhibiting function for NK cells. Thus the down-regulation of HLA that facilitates evasion of T cell recognition actually facilitates NK recognition. Clinical trials using autologous and haplo-identical (i.e., parent or sibling donor) NK cells have been conducted for several tumor types, with some promising early results. These events are depicted graphically in Figure 4.

Once factor related to the efficacy of any immune-mediated therapy that must be considered is the tumor burden at the time of treatment. Whether the intervention is T cells, NK cells, cytokines, or immune-modulating agents such as L-MTP-PE, the greatest efficacy would be expected if the intervention begins in a minimal residual disease state. All available data regarding immune therapies suggests that bulky tumors are unlikely to respond, while microscopic disease may be controlled. This fact about immune therapies needs to be considered carefully, both when clinicians and investigators are evaluating outcomes from early-phase studies and when clinician-investigators are designing phase II and phase III studies incorporating immune-based treatments. If we design trials evaluating immune approaches in advanced-disease patients, we are quite likely to discard potentially beneficial treatments because we evaluated them in the wrong cohort of patients.

## Summary and Next Steps

The desperate need for better therapies for both OS and ES is clear, given the numbers of children and young adults each year who develop treatment-resistant disease that follows a relentless, downward course. A better understanding of the biology related to the several steps of disease development and progression, especially the biology of metastasis, immune evasion, and latency/reactivation, is needed to foster new approaches. While our investigations can sometimes be informed by following the literature for common adult carcinomas, one must bear in mind the important differences between carcinomas and either OS or ES. For example, since OS is derived from mesenchymal tissues, studies of epithelial-to-mesenchymal transition (EMT) and the reverse –MET – seem unlikely to be very informative, since the tumor is mesenchymal in the first place, and the data for state changes such as EMT or MET as a part of OS and ES metastasis is not very clear. The specific signals involved in tumor dormancy in particular also are not well-characterized and may offer a new therapeutic entry point for changing the landscape of OS and ES. Three distinct parts of dormancy should be considered, as each could be its own novel therapy. First, an active signal causes OS tumor cells to arrest growth while metastasizing, which helps contribute to chemoresistance. At the same time, dormant tumor cells have an active developmental block that prevents them from pursuing a differentiation cascade. Finally, something later causes individual tumors in the metastatic niche to resume growth. The latter may be connected to angiogenesis and the cytokines of wound healing, given the association, more frequently commented upon than observed, of metastatic tumor growth beginning a few weeks after major surgery. Better understanding of these signals and pathways will enable novel therapies to be used in the manner most likely to lead to improved outcomes, such as employing immune therapies in a minimal residual disease state.

## Conflict of Interest Statement

The authors declare that the research was conducted in the absence of any commercial or financial relationships that could be construed as a potential conflict of interest.
